# Summer Precipitation Predicts Spatial Distributions of Semiaquatic Mammals

**DOI:** 10.1371/journal.pone.0135036

**Published:** 2015-08-18

**Authors:** Adam A. Ahlers, Lisa A. Cotner, Patrick J. Wolff, Mark A. Mitchell, Edward J. Heske, Robert L. Schooley

**Affiliations:** 1 Department of Natural Resources and Environmental Sciences, University of Illinois, Urbana, Illinois, United States of America; 2 Illinois Natural History Survey, Prairie Research Institute, Champaign, Illinois, United States of America; 3 College of Veterinary Medicine, University of Illinois, Urbana, Illinois, United States of America; The Ohio State University, UNITED STATES

## Abstract

Climate change is predicted to increase the frequency of droughts and intensity of seasonal precipitation in many regions. Semiaquatic mammals should be vulnerable to this increased variability in precipitation, especially in human-modified landscapes where dispersal to suitable habitat or temporary refugia may be limited. Using six years of presence-absence data (2007–2012) spanning years of record-breaking drought and flood conditions, we evaluated regional occupancy dynamics of American mink (*Neovison vison*) and muskrats (*Ondatra zibethicus*) in a highly altered agroecosystem in Illinois, USA. We used noninvasive sign surveys and a multiseason occupancy modeling approach to estimate annual occupancy rates for both species and related these rates to summer precipitation. We also tracked radiomarked individuals to assess mortality risk for both species when moving in terrestrial areas. Annual model-averaged estimates of occupancy for mink and muskrat were correlated positively to summer precipitation. Mink and muskrats were widespread during a year (2008) with above-average precipitation. However, estimates of site occupancy declined substantially for mink (0.56) and especially muskrats (0.09) during the severe drought of 2012. Mink are generalist predators that probably use terrestrial habitat during droughts. However, mink had substantially greater risk of mortality away from streams. In comparison, muskrats are more restricted to aquatic habitats and likely suffered high mortality during the drought. Our patterns are striking, but a more mechanistic understanding is needed of how semiaquatic species in human-modified ecosystems will respond ecologically *in situ* to extreme weather events predicted by climate-change models.

## Introduction

Many studies attempt to predict species’ responses to climate change [1] and most focus on changes in geographic distributions [1–3] and potential *in situ* evolutionary adaptation [4–5]. However, many animal species will need to make ecological adjustments within geographic range interiors, such as altering habitat selection, to deal with increased environmental stochasticity. These responses should have consequences for species persistence and may be affected by human alterations of the landscape. To understand how populations will respond to predicted climate-change scenarios, a necessary step is to investigate temporal variation in species occurrences relative to a range of weather conditions.

Climate change is increasing the variability of precipitation and frequency of extreme flooding and drought events [6–7]. Species obligately associated with wetland and stream habitats are particularly at risk due to extreme fluctuations in water levels. As these climate-sensitive habitats become less stable, species dispersal [8], recruitment [9], and survival [9–12] could be compromised. Semiaquatic species might need to move to other suitable habitat patches to persist during times of environmental stress, but moving across terrestrial areas can be costly [13–14], especially in regions where agriculture and urbanization have destroyed linkages and reduced connectivity.

American mink (hereafter mink; *Neovison vison*) and muskrats (*Ondatra zibethicus*) are semiaquatic mammals that may be sensitive to increased variation in precipitation events. Both species are widely distributed throughout North America and are obligately associated with aquatic habitats, although the degree of this association differs between species. Muskrats are chiefly herbivores and most of their diet consists of wetland vegetation. Space use by muskrats is mostly restricted to the stream edge and movements >3 m away from water are rare [15]. Mortality from predation is high during drought conditions and likely due to the limited mobility of muskrats away from water and reluctance to leave established home ranges [16–17]. Additionally, increased flooding can reduce survival of young [18]. Mink are generalist predators that forage in aquatic and terrestrial habitats [19]. When aquatic prey (e.g., fish, amphibians, and crayfish) are unavailable, mink will forage more frequently in terrestrial areas [20], which could expose them to elevated risks. However, mortality risk for mink in terrestrial versus stream habitats is unknown.

We used 6 years of presence-absence data spanning years of record-breaking floods and drought to assess how mink and muskrats respond to conditions predicted to increase under climate-change scenarios. Specifically, we tracked annual changes in site occupancy for mink and muskrats in response to variable summer precipitation. We also radiomarked individuals to assess mortality risk for both species in terrestrial habitats as activity in these areas may become more common with increasing environmental variability. In our study system, > 90% of wetlands have been drained to accommodate agricultural production [21], thus limiting both species’ distributions primarily to flashy streams and rivers. Species occurring in these human-dominated landscapes may be at an increased risk owing to the synergistic effects of habitat loss and climate change [22–23].

We hypothesized that mink and muskrat populations in our region would be sensitive to summer precipitation because droughts reduce habitat quality for semiaquatic mammals in streams. Droughts lower water levels and persistence of flow, thus reducing the protection from predation, and availability of aquatic prey, afforded by deeper water. Thus, we predicted habitat occupancy for both species would be correlated positively with summer precipitation across years. We assumed differences in annual occupancy rates for species reflected underlying patterns of abundance [24–25]. Because muskrats are more tightly associated with streams than are mink, we predicted negative effects of droughts would depress muskrat abundance more than mink abundance. We also assessed the extent of terrestrial habitat use by mink and muskrats and predicted mortality risk would be greater in terrestrial habitat than in stream habitat.

## Materials and Methods

### Study area

**Fig 1:**
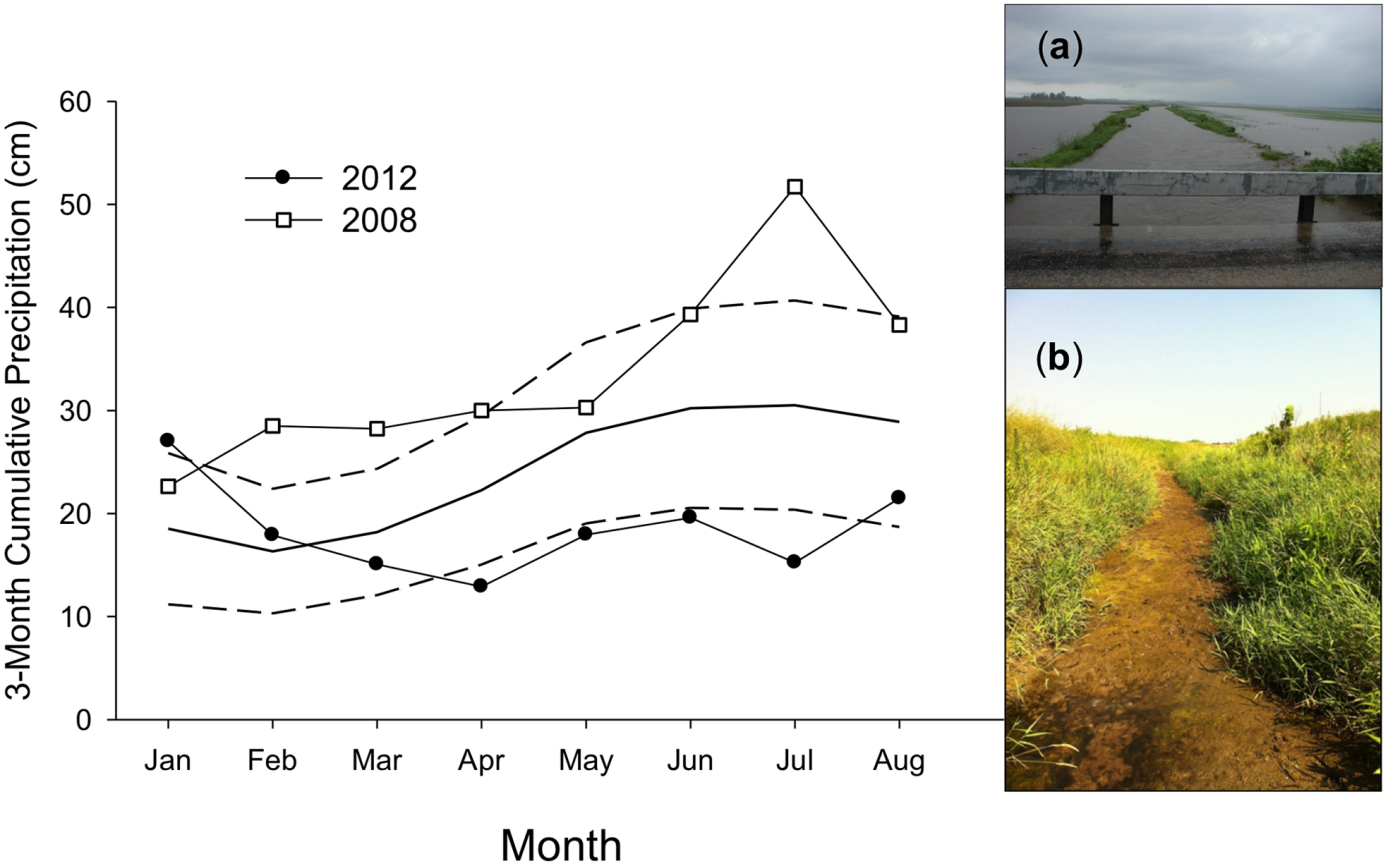
Three-month cumulative precipitation (sum of current month and the previous two months) in Urbana, Illinois prior to occupancy surveys for mink (*Neovison vison*) and muskrat (*Ondatra zibethicus*). Mean (solid line) ± 1 SD (dashed line) represent the historical 3-month cumulative precipitation (1889–2012). Photographs are from the same stream segment during (a) 2008 and (b) 2012.

Our study was conducted in east-central Illinois, USA. (40°12’N, 88°26’W) in a region that is intensely farmed and highly fragmented. This region has a humid continental climate with temperatures ranging from -8.5 to 30.0°C and experiencing ~175 cm of precipitation annually. Currently, 85% of the landscape is dedicated to corn (*Zea mays*, 45%) and soybean (*Glycine max*, 40%) production, and wetlands cover only 0.9% of the landscape [21, 26]. Consequently, small streams and agricultural ditches that form narrow riparian corridors represent the primary habitat for semiaquatic mammals in the region. These habitats have dynamic flow regimes tied to local precipitation events [27–28]. Climate models predict this region will experience a significant increase in the frequency of summer drought and spring flooding events [29–30], thus increasing flow variability and potentially affecting habitat quality for semiaquatic species. In 2008, the region experienced the 2^nd^ wettest year on record [31]. In 2012, the region experienced the 2^nd^ driest January—July period on record ([32]; Fig 1).

### Sampling design

We used a stratified-random sampling design to select 90 survey sites along riparian areas (S1 Table). All survey sites were located on property owned or controlled by private individuals, municipalities, land trusts, or state agencies. We obtained permission from all landowners prior to surveys. Contact information for the owners of these properties can be obtained from the corresponding author (AAA). Fifty percent of the sites (*n* = 45) were randomly chosen within a 2-km radius of incorporated cities (population size >2500), and the remainder (*n* = 45) were randomly chosen outside of this buffer. Each site was a 200-m stretch of wadeable stream (ranging from 1^st^ to 5^th^ order in size) and represented a potential resource patch for both mink and muskrats [33–36]. Median nearest-neighbor distance between sites was 2.5 km (range = 0.5–22.8 km).

Sites were surveyed by trained, independent observers for presence of mink (tracks and scat) and muskrats (tracks, scat, clippings and burrows) using a removal-design framework [37] from July to October, 2007–2012. Each site was surveyed by two independent observers simultaneously, with each observer beginning their survey on opposite ends of the stream segment during each site visit (two surveys during one site visit; 35). Initially (2007–2008), surveys were developed to assess muskrat occupancy (removal design based on muskrat sign) and each site was surveyed twice for both species but not revisited if muskrat sign was detected (2 surveys). If muskrat sign was not detected during the first site visit, we conducted an additional site visit to survey for both species for a maximum of four surveys per site [35]. From 2009–2012, if mink sign was not found during the first site visit (removal design based on mink sign), we conducted an additional site visit yielding a maximum of four surveys per site. For each year, we limited the time between site visits to ≤ 10 days. We randomly reduced the number of sites from 90 to 60 in 2009–2012 due to logistical constraints. The occupancy modeling approach that we used efficiently handles missing observations as created by our mixed removal design and reduction in number of sites [38]. To reduce risk of sign being washed away by rain or rising water, we waited >2 days to survey sites that had experienced weather events with ≥1 cm of precipitation. Overall, we conducted 1196 surveys (2007 = 276; 2008 = 282; 2009 = 130; 2010 = 160; 2011 = 162; 2012 = 186) that spanned ~239 km of wadeable stream.

### Site occupancy analysis

We fit multi-season models using Program PRESENCE 6.9 to derive model-averaged annual estimates of site occupancy for each species given unique detection histories [38]. For each model, we held initial occupancy (*Ψ*_2007_) constant, let colonization (γ) and extinction (ε) vary by year, and varied survey-specific covariates for species detection (*p*). Because the goal of this analysis was to derive robust estimates of annual habitat occupancy for the region, we were not concerned with site-specific habitat variables important for individual site occupancy and turnover. Potential detection covariates included survey date, recent rainfall, observer effects, and amount of trackable surface along the stream edge [35–36]. Additionally, we considered the amount of debris within the stream (emergent rocks and logs used for scat deposition by muskrats) in models of muskrat detection [35]. Survey date (Date) was the day of the year when the survey was conducted (1–365). We acquired rainfall data from the Illinois State Water Survey (station 118740; Urbana, IL) and summed precipitation for 7 days prior to each survey (Rain). Observer effects (Observer) were coded in relation to a reference observer ([38]: pp. 117–118). Thirteen observers conducted surveys from 2007–2012. To avoid overparameterization of models, we grouped observers based on survey effort and modeled six total observers. We visually estimated the percent of trackable surface along the stream edge (Sandbar) starting in 2008; we did not measure ‘Sandbar’ during 2007 surveys. Because ‘Sandbar’ is an important detection covariate for mink [36], we estimated values for 2007 *a posteriori* for each site using mean Sandbar values for each site from 2008–2012. Average Sandbar indices for each site were highly correlated between years (mean Pearson correlation coefficient = 0.60, range = 0.49–0.80, *P* < 0.0001). We quantified the relative amount of debris within each site (Debris) on a scale of 0–5, with 0 = no debris and 5 = ≥1 piece of debris every 10 m. We used Akaike’s Information Criterion corrected for small sample sizes (AIC_c_) to rank models within the candidate set for each species. Additionally, we used the Akaike weights (ω) to derive model-averaged estimates of annual site occupancy [38] for mink and muskrats using all models from each species candidate set.

### Precipitation

We used generalized linear models (PROC GENMOD, distribution = normal, link = identity; [39]) to assess the importance of summer precipitation to annual site occupancy of mink and muskrats. We summed the 3-month cumulative rainfall prior to occupancy surveys (May, June and July) for 2007–2012 (station 118740; Illinois State Water Survey) and used this value as a proxy for regional summer precipitation. The weather station was centrally located in our study area (Urbana, IL) and recorded daily precipitation representative of our sites. We used a logit transformation for our response variables (model-averaged estimates of annual site occupancy for mink and muskrats) to meet linear model assumptions [40] and calculated a pseudo *R* ^2^ (1- [deviance of fitted model/deviance of intercept-only model]) to assess each model’s goodness-of-fit.

### Tracking space use and survival

We radiomarked and tracked mink to assess the frequency of space use and mortality risk in terrestrial areas, and compare these results to our previous studies of muskrat space use and survival [15, 28]. We captured mink using baited (salmon or sardines) Tomahawk live traps (Model 202) attached to floating raft platforms [41] from 2009 to 2013. Traps were checked daily, refreshed with bait as needed, and closed during periods of inclement weather. We transported animals to a sterile surgical laboratory at the Veterinary Teaching Hospital at the University of Illinois (Urbana, Illinois, USA) immediately after capture. We surgically implanted radio transmitters into the peritoneal cavities of 34 mink using methods similar to those outlined in our previous studies [15, 28, 42]. Prior to surgery, mink were premedicated with atropine (0.20 mg/kg), dexmedetomidine (0.25 mg/kg), and butorphanol (0.30 mg/kg). We induced surgical aesthesia via facemask with isoflurane (5% for induction and maintained between 1–3% throughout procedure) while simultaneously administering and maintaining oxygen (0.60–1.00 l/min). We fitted smaller mink (<500 g) with 14-g internal transmitters (Model 1215; Advanced Telemetry Systems, Insanti, Minnesota, USA) and larger mink (≥500 g) with 23-g internal transmitters (Model 1230). Transmitters were equipped with mortality sensors that increased pulse rate when inactive for ≥ 8 hours allowing us to quickly retrieve the carcass and determine location and cause of mortality. After transmitters were implanted, we administered atipamazole (2.50 mg/kg) to reverse the sedative effects of dexmedetomidine and meloxicam (0.20 mg/kg; post-operative analgesic). Additionally, we administered penicillin (0.10 ml) to limit post-operative infections. We monitored recovering animals for approximately 2 hours (after gaining all righting reflexes) and returned them to the site of capture.

We relocated mink using a combination of triangulation (when mink were active) and homing (when mink were inactive). Prior to the study, we used hidden test transmitters (n = 10) and determined triangulation error was minimal ($\bar{x}$ = 16.6 m; SD = 14.3). We attempted to relocate individual mink at least once per week. Detailed descriptions of muskrat capture, marking, and radiotracking methods are described in our previous studies along with a comprehensive analysis of muskrat space use and mortality [15, 28]. We did not include endangered or threatened species in any part of our study and all methods and procedures were approved by the University of Illinois Animal Care and Use Committee (Protocols 07105 and 12190) and met guidelines of the American Society of Mammalogists [43].

A detailed analysis of habitat selection and survival by mink and muskrats is beyond the scope of this paper. However, we present a coarse assessment of terrestrial habitat use based on the proportion of relocations for radiomarked mink at various distances away from the stream edge and compare this to previously published space-use patterns for muskrats [15]. We only considered mink with ≥ 25 locations (*n* = 20) for this analysis. To determine the mortality risk for mink (*n* = 34) and muskrats (*n* = 27; 28) in terrestrial habitat, we determined the likelihood of mortality in relation to distance from the stream edge. For mink, we tested for differences in the distributions of mortality locations and telemetry locations in relation to distance from the stream edge with a two-sample Kolmogorov-Smirnov test. For muskrat, we could not statistically test for potential distribution differences because of the small number of observed mortalities in terrestrial areas and no observed movements in terrestrial habitat (see Results).

## Results

**Fig 2:**
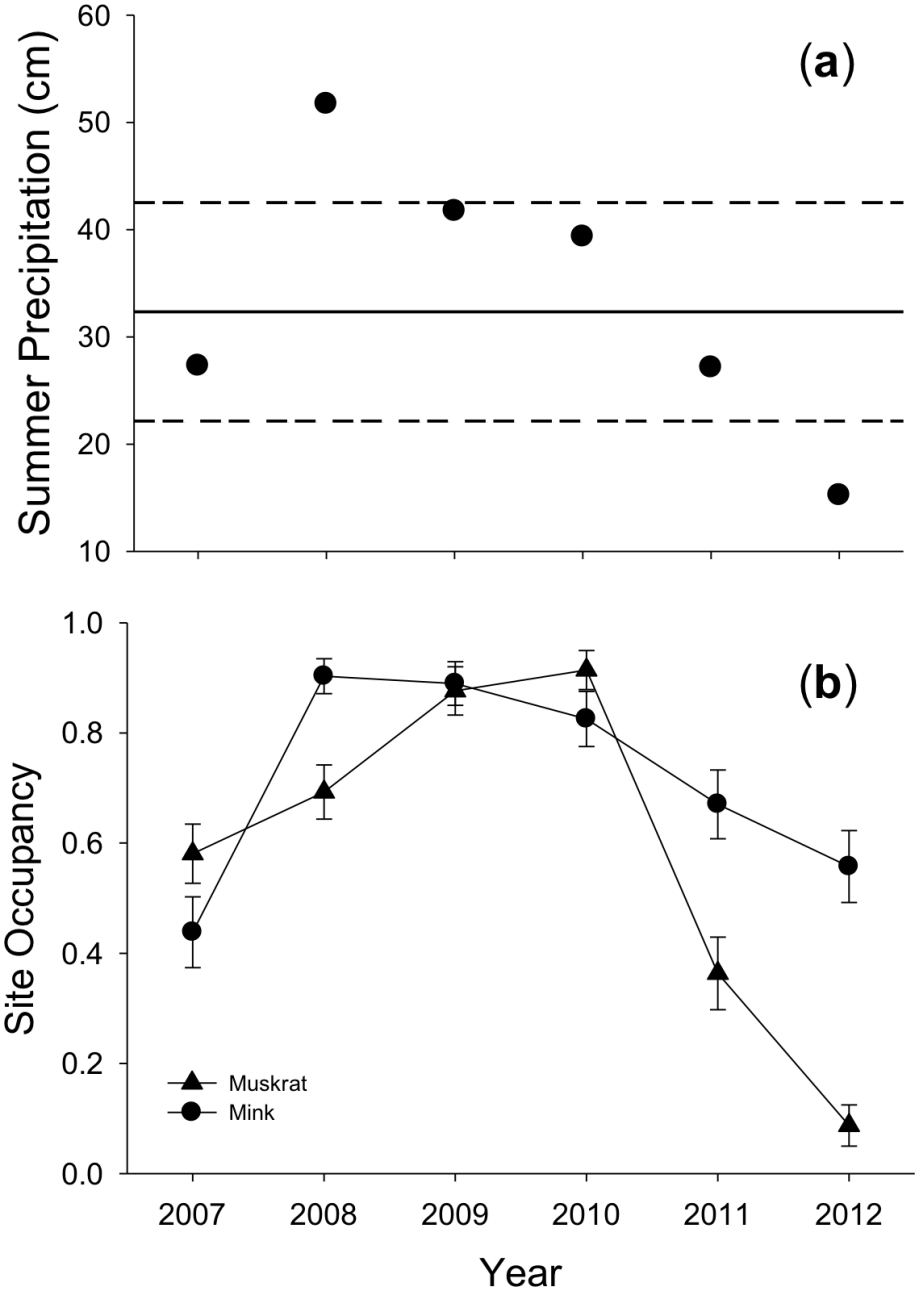
Trends in (a) summer precipitation and (b) site occupancy dynamics of mink (*Neovison vison*) and muskrats (*Ondatra zibethicus*) in Illinois, USA from 2007–2012. Summer precipitation (sum of May, June, and July) for each year is compared to the 123-year mean (solid line) ± 1 SD (dashed line) for the same period. Estimates of site occupancy (± 1 SE) are model-averaged and corrected for imperfect detection.

Based on a 123-year record of precipitation, the average summer precipitation for our region was 32.3 cm (SD = 10.2; Fig 2a). During our study, summer precipitation (cm) was extremely variable among years (Fig 2a): 2007 = 27.3; 2008 = 51.7; 2009 = 41.7; 2010 = 39.3; 2011 = 27.2; 2012 = 15.2. Summer precipitation was high during a year of record-breaking floods (2008 = 51.8 cm) and low during a year with widespread drought (2012 = 15.2 cm).

Our ability to detect muskrat sign was negatively affected by the amount of rain 7 days prior to surveys (β = -0.0897, SE = 0.05; Table 1). Models including ‘Rain’ had the most support among all models of muskrat detectability (Σω = 0.67). Although four other models were competitive, none had a substantially better model fit than the top-ranked model (Table 1). Thus, we considered the variables ‘Sandbar’, ‘Date’, and ‘Debris’ non-informative [44–45]. The intercept-only model also was among the top-ranked models but it had a reduced model fit compared to our best model.

**Table 1:** Ranking of multi-season models for detection (*p*) of riparian muskrats (*Ondatra zibethicus*) and American mink (*Neovison vison*) in Illinois, USA from 2007–2012.

Model	ΔAIC_c_	ω	K	-2LogLike
**Muskrat**				
*Ψ*(.), γ(2008–2012), ε(2008–2012), *p*(Rain)	0.00	0.18	13	1046.77
*Ψ*(.), γ(2008–2012), ε(2008–2012), *p*(Rain + Sandbar)	1.26	0.09	14	1046.03
*Ψ*(.), γ(2008–2012), ε(2008–2012), *p*(Rain + Date)	1.49	0.08	14	1046.26
*Ψ*(.),γ(2008–2012), ε(2008–2012), *p*(Rain + Debris)	1.55	0.08	14	1046.26
*Ψ*(.), γ(2008–2012), ε(2008–2012), *p*(.)	1.93	0.07	12	1050.70
**Mink**				
*Ψ*(.), γ(2008–2012), ε(2008–2012), *p*(Observer + Sandbar)	0.00	0.37	18	1230.99
*Ψ*(.), γ(2008–2012), ε(2008–2012), *p*(Observer + Sandbar + Date)	0.77	0.25	19	1229.76
*Ψ*(.), γ(2008–2012), ε(2008–2012), *p*(Observer + Sandbar + Rain)	0.98	0.22	19	1229.97
*Ψ*(.), γ(2008–2012), ε(2008–2012), *p*(Observer + Sandbar + Date + Rain)	1.99	0.14	20	1228.98
*Ψ*(.), γ(2008–2012), ε(2008–2012), *p*(.)	42.03	0.00	12	1285.02

ΔAIC_c_ = difference between model AIC_c_ and lowest AIC_c_. ω = Akaike weights. K = number of estimable parameters. -2LogLike = twice the negative log-likelihood. For both species, we present all models with ΔAIC_c_ ≤ 2, along with the base model. The base model includes parameters for initial occupancy in 2007 [*Ψ*(.)], annual colonization [γ(2008–2012)], annual extinction [ε(2008–2012)], and constant detection probability [*p*(.)]. Detection covariates include rain 7 days prior to survey (Rain), percentage of trackable surface (Sandbar), day of year site was surveyed (Date), amount of debris (Debris), and observer conducting survey (Observer).

Four models of mink detectability were competitive (ΔAIC_c_ ≤ 2; Table 1). Each model contained the variables ‘Observer’ and ‘Sandbar’. Our second- and third-ranked model also included the effects of ‘Date’ and ‘Rain’, respectively, and the fourth-ranked model included the additive effects of ‘Observer’, ‘Sandbar’, ‘Date’, and ‘Rain’ (Table 1). In concordance with past research [36], our ability to detect mink sign was affected by observer variability (range of βs = -1.8288–0.8105), positively related to amount of trackable surface (β = 0.0031, SE = 0.0014) and survey date (β = 0.0036, SE = 0.0006), and negatively related to amount of rain 7 days prior to surveys (β = -0.0417, SE = 0.0466).

**Fig 3:**
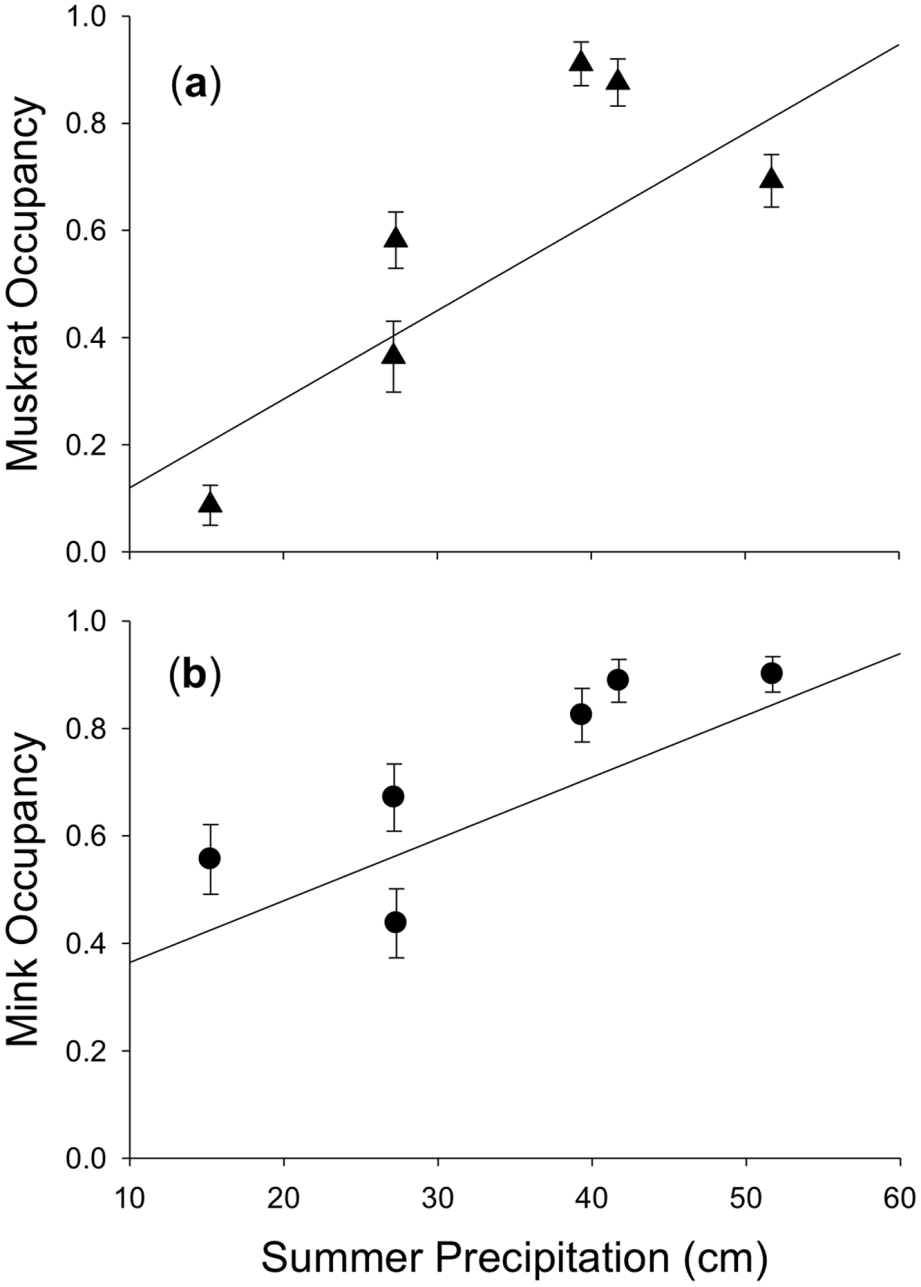
Relationship between site occupancy by (a) muskrat, and (b) mink and 3-month precipitation (May, June, and July) from 2007–2012 in Illinois, USA. Estimates of site occupancy (± 1 SE) are model-averaged and corrected for imperfect detection.

Model-averaged estimates of site occupancy by muskrats varied substantially among years (Fig 2b): 2007 = 0.58 (SE = 0.05), 2008 = 0.69 (SE = 0.05), 2009 = 0.88 (SE = 0.04), 2010 = 0.91 (SE = 0.04), 2011 = 0.36 (SE = 0.07), 2012 = 0.09 (SE = 0.04). Occupancy rates were higher in years when summer precipitation was above the 123-year mean (2008, 2009 and 2010), and occupancy rates were lower when summer precipitation was below the 123-year mean (2007, 2011 and 2012). The estimated proportion of sites occupied by muskrats each year was positively associated with summer precipitation (β = 0.1045; *P* = 0.0016; pseudo *R* ^2^ = 0.62; Fig 3a).

Our model-averaged estimates of annual site occupancy by mink also varied among years (Fig 2b): 2007 = 0.44 (SE = 0.06), 2008 = 0.90 (SE = 0.03), 2009 = 0.89 (SE = 0.04), 2010 = 0.82 (SE = 0.05), 2011 = 0.67 (SE = 0.06), 2012 = 0.56 (SE = 0.06). Mink were widely distributed during years with above-average precipitation (2008, 2009 and 2010). Estimated occupancy rates were lower during years with below-average precipitation (2007, 2011 and 2012), but remained moderately high in 2011 following three relatively wet years and did not decline to the extent observed for muskrats during the extreme drought of 2012 (Fig 2). As predicted, the proportion of sites occupied by mink each year also was positively related to summer precipitation (β = 0.0681; *P* < 0.0001; pseudo *R* ^2^ = 0.77; Fig 3b).

**Fig 4:**
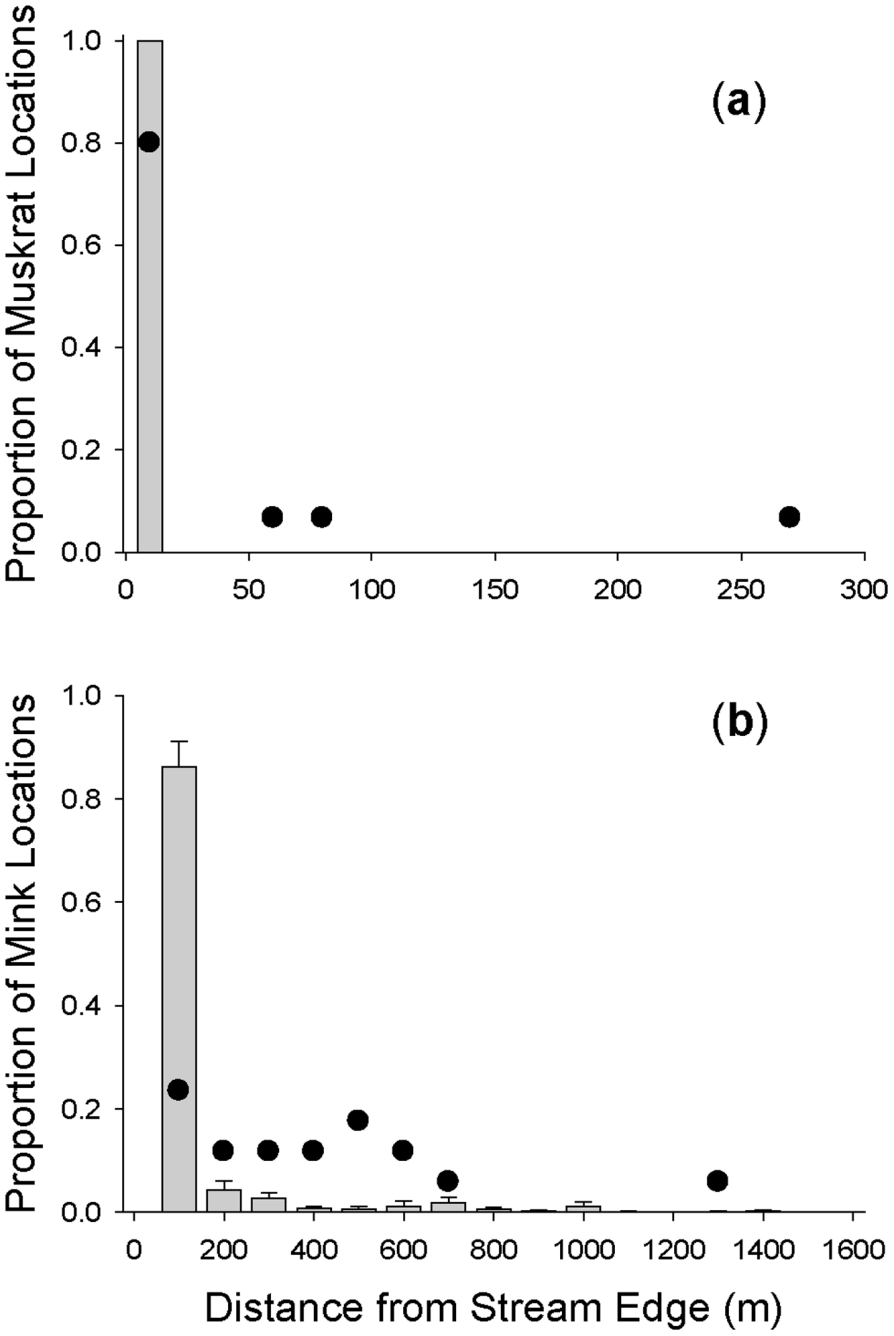
Bars indicate the proportion of locations (mean + 1 SE) of radiomarked (a) muskrat (*n* = 26) and (b) American mink (*n* = 20) in relation to distance from the stream edge. Locations are grouped into 10-m bins for muskrats and 100-m bins for mink. Dark circles represent the proportion of known-fate mortalities in relation to distance from the stream edge. We adapted space-use and mortality data from our previous studies (15, 28). Muskrat movements never exceeded > 3 m from the stream edge. Note differences in scale of *x* axis for (a) and (b).

For the 20 mink for which we had sufficient movement data to assess extent of habitat use in terrestrial areas, each individual was relocated an average of 102 times (SE = 10.27; range = 25–192) for a total of 2035 locations (Movebank.org Data Repository, dx.doi: 10.5441/001/1.gd686078). The distribution of mink mortalities differed from the distribution of telemetry locations in relation to distance from the stream edge (*D* = 0.62; *P* < 0.0002). On average, mink were relocated >100 m from the stream edge only 14% of the time (Fig 4b; S2 Table). In contrast, of 17 known-fate mortalities (seven road kill, six predation, three poisoning and one disease), 76% (*n* = 13) occurred when mink were >100 m from the stream edge (Fig 4b; S2 and S3 Tables). Our previous studies of muskrat space use and survival found that muskrats rarely used upland habitat and were never relocated > 3 m from the stream edge (Fig 4a; 15). Of 15 known-fate mortalities, 80% (*n* = 12) occurred along the stream edge and were attributed to mink predation (Fig 4a; 28). We recovered the other 3 muskrat carcasses in or around coyote (*Canis latrans*) and red fox (*Vulpes vulpes*) burrows > 50 m from the stream edge [28]. One canid-related mortality occurred while the muskrat was displaced into a corn field during a flooding event. Because we did not detect muskrat movements > 3 m away from the water’s edge, all mortalities attributed to canid mortality likely occurred along the stream edge and carcasses were transported back to active canid burrows [28].

## Discussion

Annual occupancy of stream segments by mink and muskrats was strongly related to summer precipitation. Occupancy rates for both species were higher during years with above-average precipitation than years with below-average precipitation (Fig 2). This contrast was especially clear for muskrats; estimated annual occupancy rates were > 10 times lower (from 0.91 to 0.09) during the severe drought of 2012. Increased frequency of summer droughts is predicted by climate models for the Midwestern USA [29], and the patterns observed in 2012 may therefore be a harbinger for semiaquatic mammals.

Mink and muskrats were widely distributed during years with above-average precipitation. Higher water levels due to increased precipitation likely provided more suitable habitat and increased connectivity between areas of high-quality habitat for both species. Higher water levels also may have provided escape routes from terrestrial predators and lowered predation risk for both species. Previous research demonstrated that site occupancy for mink and muskrats is correlated positively with water depth [35–36, 46]. Furthermore, the probability of vacant sites being recolonized by both species is positively related to water depth [35–36]. Conversely, mink and muskrat occupancy rates were lower during years of below-average precipitation. Low water levels can limit available resources and reduce overall body condition of muskrats resulting in increased mortality [47]. Muskrats also are susceptible to increased predation risk during drought because their locomotion is more limited on land than in water, and the openings of their burrow dens may be exposed as streams dry [17]. Despite this deterioration in habitat quality during drought, muskrats are typically reluctant to leave their home ranges to find other suitable habitat [16], and this effect may be exacerbated in areas where habitat loss has reduced spatial connectivity.

Investigations of how mink respond behaviorally to drought are lacking. However, reduced occupancy rates during years of below-average precipitation suggest mink are foraging in terrestrial habitat away from the stream edge. We think the decline in occupancy for mink partly represents increased use of alternate habitats rather than just mortality because mink are not as constrained to aquatic habitats in our region as are muskrats. All telemetry locations of muskrats occurred within 3 m of stream banks [15]. In contrast, 14% of telemetry locations of mink occurred >100 m from stream banks (Fig 4b), revealing more flexibility by mink in habitat use. The switch to terrestrial habitats may come with increased mortality costs, however, as mortality risk was disproportionately greater for mink when moving through terrestrial areas (Fig 4b). Thus, the greater mobility of mink may allow them to exploit secondary habitats during droughts, reducing their susceptibility to degradation of stream habitats relative to muskrats in the short term. However, if climate change increases the frequency of droughts [7], increased use of more risky habitats by mink should eventually reduce survival rates and affect population dynamics. Unfortunately, we have insufficient data on mortality by muskrats or mink during the drought year to evaluate this hypothesis directly.

Although the patterns are clear, we acknowledge a caveat associated with our interpretations. We cannot directly link discrete flooding and drought events during our study to changing climate. Nevertheless, these extreme events will be more common in the future [6–7, 29–30]. Contemporary climate models suggest severe and widespread drought this century [7]. Species obligately associated with drought-sensitive habitats will be most at risk. Thus, population patterns associated with observed climate-driven events should mimic those during future climate-change conditions.

In Canada, mink and muskrats represent a classic predator-prey system in which mink populations exhibit a lagged numerical response to changes in muskrat abundances [48–50]. However, there is significant geographic variation in the strength of this predator-prey relationship [48, 50]. Mink and muskrat population dynamics in Canada may be partially affected by the spatial variability in winter precipitation [51]. Additionally, differences in predator-prey interaction strength may be partially attributed to spatial variability in prey richness across Canada [52]. In our region, habitat occupancy for mink and muskrats was strongly related to summer precipitation (Fig 3). This correlation suggests environmental variability affects populations of both species similarly and possibly decouples any classic predator-prey relationship. Mink diet in this region is diverse and seasonal occurrence of mammals in mink scats were always <50% of the percentage volume of sampled scats [53]. Increased diversity of mink prey in this region may release muskrats from the specialized predation pressure necessary for cyclic population dynamics [54]. In our study, synchrony of mink and muskrat populations (without a time lag) and their sensitivity to summer precipitation suggest these populations are largely limited by external forcing.

Many semiaquatic species may be negatively affected by the synergistic effects of habitat loss and climate change. Interactions between these stressors can depress population densities and reduce species diversity [23]. Moreover, increases in the frequency and intensity of regional flooding and drought can potentially synchronize population dynamics of species at large spatial scales—especially habitat specialists occurring in homogenous agricultural landscapes [22, 55]. For instance, intensively farmed landscapes can function as habitat sinks for common frog (*Rana temporaria*) populations during extreme drought compared to landscapes retaining some heterogeneity [55]. Additionally, populations of platypus (*Ornithorhynchus anatinus*), another obligate wetland species, are threatened by both increasing thermal stress and habitat loss due to climate change and increased irrigation demands for agriculture [56].

In many ecosystems worldwide in which most wetland habitat has been converted to agriculture, the primary remaining habitats for semiaquatic species are small, flashy streams. Because habitat suitability for these species is generally linked with water availability, increased variability in precipitation should drive spatial and temporal variation in habitat quality. A more mechanistic understanding is urgently needed of how extreme weather events, like those observed in our study and predicted under climate-change models, will affect populations of semiaquatic species in human-modified environments.

## Supporting Information

S1 Table The locations and occupancy status of study sites used to determine annual habitat occupancy patterns of American mink (*Neovison vison*) and muskrats (*Ondatra zibethicus*) in east-central Illinois, USA. Site locations were recorded in the North American Datum coordinate system (WGS84 UTM Zone 16N) and represent the center of each 200-m stream segment. Site = assigned site name; Northing and Easting = Cartesian coordinates for the center of each study site; Muskrat = naïve muskrat occupancy status at a particular site during a given year (1 = sign found at least one survey at site, 0 = sign not found during any survey at site,— = site not surveyed); Mink = naïve mink occupancy status at a particular site during a given year (1 = sign found at least once at site, 0 = sign not found at site,— = site not surveyed).
(XLSX)

S2 Table Distribution of American mink (*Neovison vison*) locations and mortality events in relation to distance from the stream edge (m). All locations and mortality events are grouped into 100 m bins.
(XLSX)

S3 Table Locations, dates, and causes of 17 American mink (*Neovison vison*) known-fate mortalities in east-central Illinois, USA. Mortality = individual mink mortality; Northing and Easting = Cartesian coordinates for the mortality location; Date = date the mortality was recorded; Cause = known cause of mortality.
(XLSX)
